# Patient and Physician Satisfaction with Telemedicine in Cancer Care in Saskatchewan: A Cross-Sectional Study

**DOI:** 10.3390/curroncol29060309

**Published:** 2022-05-27

**Authors:** Hurria Gondal, Tahir Abbas, Heather Choquette, Duc Le, Haji Ibraheem Chalchal, Nayyer Iqbal, Shahid Ahmed

**Affiliations:** 1 Division of Oncology, College of Medicine, University of Saskatchewan, Saskatoon, SK S7N5E5, Canada; hurriagondal@outlook.com; 2 Saskatchewan Cancer Agency-Saskatoon Cancer Centre, University of Saskatchewan, Saskatoon, SK S7N4H4, Canada; duc.le@saskcancer.ca (D.L.); nayyer.iqbal@saskcancer.ca (N.I.); 3 Community Oncology Service, Saskatchewan Cancer Agency, Regina, SK S4T7T1, Canada; heather.choquette@saskcancer.ca; 4 Saskatchewan Cancer Agency-Allan Blair Cancer Centre, University of Saskatchewan, Regina, SK S4T7T1, Canada; haji.chalchal@saskcancer.ca

**Keywords:** telemedicine, telehealth, cancer care, rural residence, patient satisfaction

## Abstract

Background: Telemedicine is a useful tool that connects patients to their care team remotely and improves access to medical care for rural residents. This study aimed to determine the telemedicine experience of both rural patients with cancer and their physicians, and to explore factors associated with a positive patient experience. Methods: In this cross-sectional study, cancer patients and physicians in Saskatchewan completed a paper-based survey composed of 32 items or an electronic survey of 18 items, respectively. Logistic regression analysis was performed to assess patient satisfaction in relation to various sociodemographic and cancer-related factors. Results: Overall, 25 physicians and 165 patients participated in the study. Among the physicians, 94% were confident in their telemedicine assessment, 58% agreed that telemedicine improved clinical efficiency, and 73% agreed that doctor–patient rapport was unimpaired with telemedicine. Of 165 patients, 61% had used telemedicine for the first time, 81% felt that their needs were met, 83% were satisfied with the quality of their care, and 88% had a positive experience. Overall, 83% patients vs. 45% physicians preferred telemedicine to a face-to-face clinic visit (*p* = 0.005). On univariate analysis, patients ≥ 65 years old had a greater positive telemedicine experience compared to patients < 65 years old (odds ratio 4.1 [1.2–13.8], *p* = 0.02). Conclusion: Both patients and physicians have a high rate of positive experiences with telemedicine. However, patients have a higher preference for telemedicine over face-to-face visits compared to physicians. In addition, elderly patients have more positive telemedicine experiences compared to younger patients.

## 1. Introduction

Telemedicine provides remote and timely services to patients who live far away from main health care facilities and who would otherwise need to travel long distances for periodic assessment and management of their illness [1,2,3]. It relies on telecommunication and videoconferencing technology, and provides an alternative to face-to-face appointments. By enabling assessment of patients in their familiar surroundings, telemedicine improves access to health care [1,2,3]. Telemedicine services use a variety of technological means to support clinical care [4,5]. In its simple form, telemedicine is a real-time videoconferencing session that shares some characteristics of a traditional in-person appointment without requiring long-distance commuting. 

On average, patients from rural communities in North America and Australia travel two to three hours to access health care that is not readily available close to their home [6,7,8]. Vulnerable patients, including cancer patients, elderly patients, patients with comorbid illnesses or limited mobility, and patients with low socioeconomic factors, are placed under the additional burden of travelling for their medical care when they live rurally. The long distance and related costs create inequality and barriers for patients to access health care, a determinant of health [9]. Cancer patients require periodic follow-up visits for assessment and treatments, which could be challenging for rural residents and their family. The arrival of new technology has changed the way cancer care is delivered, introducing the option of using a more technologically based virtual visit instead of an on-site appointment with a physician. Despite its advantages, telemedicine has been used more commonly in the primary care setting than in specialty medicine. However, since 11 March 2020, with the declaration of the COVID-19 pandemic by the World Health Organization (WHO), the use of telemedicine and videoconferencing has become an integral component of patient care in specialty medicine and urgent care settings, in order to reduce viral transmission and limit interruption in the delivery of ambulatory services [10,11,12]. 

Although the use of telemedicine has been associated with overall satisfaction among patients, few studies have evaluated the telemedicine experience of rural cancer patients and their oncologists and explored the socioeconomic and clinical factors associated with better satisfaction [13,14,15]. The present study aimed to explore patients’ and physicians’ satisfaction with telemedicine and to assess patients’ satisfaction in relation to various sociodemographic and cancer-related factors, including age, gender, race, income, and education. The study was conducted about a year before the declaration of the COVID-19 pandemic. As the COVID-19 pandemic has altered the model of care in oncology, and telemedicine has been systemically adopted in cancer care by most jurisdictions, the present study provides useful information on baseline satisfaction with telemedicine among rural cancer patients during pre-COVID period. 

## 2. Methods

This cross-sectional study was approved by the Behavioral Ethics Board of the University of Saskatchewan. Saskatchewan is a geographically large population with a land area of 588,239.21 km^2^ and a population density of 1.8 persons per km^2^, which is well below the national population density of 3.7 persons per km^2^ [16]. It is home to two major northern and southern cancer centers, with many community centers providing oncological care. Cancer patients and their treating physicians in the province of Saskatchewan, Canada, completed a paper-based survey consisting of 32 items or an electronic survey of 18 items, respectively. The surveys were conducted at a number of remote locations connecting the patients to their oncologists at the two major cancer centers in Saskatoon and Regina from March 2019 to May 2019 over a period of three months. Adult patients aged ≥ 18 years with a history of cancer who had used telemedicine during the study period were eligible. 

Consecutive sampling was used, and all adult patients aged ≥ 18 years with cancer who used telemedicine during the study period were offered the opportunity to participate in the survey. Patients who participated in telemedicine underwent a preliminary assessment by a nurse at a health care facility that offered telemedicine closer to their home. The clinical data were transmitted prior to the meeting for an oncologist to review. The patient and their family, and in some cases primary care physicians, were connected to their treating oncologist and nurse via a real-time videoconferencing session. The survey was developed locally by adapting other surveys on telemedicine [17]. The patient survey included questions related to demographic and socioeconomic variables. A six-point Likert score (1 = strongly disagree, 6 = strongly agree) was used for most survey questions. For the purpose of analysis, responses that agreed or strongly agreed were considered positive. The patient survey also contained an open-ended section for comments. A thematic analysis of comments was not performed. Using one of Statistics Canada’s definitions for rural populations, rural residence was defined as residing in non-metropolitan regions, including urban settlements with populations of fewer than 50,000 people, and regions with no urban settlements (where “urban settlements” are defined as places with populations of 2500 or more) [18]. 

Logistic regression analysis was performed to assess the association between self-reported clinical and social factors and a positive telemedicine experience. The following variables were examined with respect to their relationship with a positive telemedicine experience: age (≥65 or <65 years), gender, race (Caucasian vs. non-Caucasian), work status (retired/unemployed/disability vs. full time/part time/self-employed), marital status (married/common law vs. single/widowed/separated), presence of dependent family member, education (university vs. non-university), income (≥80 K vs. <80 K), underlying cancer (solid-organ cancer vs. hematological malignancy), treatment goal (curative vs. no curative treatment), center (Saskatoon vs. Regina), surgery, radiation, and chemotherapy. A two-sided *p*-value of < 0.05 was considered statistically significant. As per prespecified criteria, if more than one variable had a *p* value of < 0.05 on univariate analysis, they were examined in a multivariate model to assess their independent association with telemedicine satisfaction. The likelihood-ratio test and *t*-test were applied to the multivariate model to determine whether the addition of independent variables of interest significantly improved the prediction of satisfaction with telemedicine in the model. SPSS version 24.0 (IBM, Armonk, NY, USA) was used for statistical analysis.

## 3. Results

### 3.1. Participants’ Characteristics

Overall, 25 physicians and 165 patients responded to the survey over the study period. Among physicians, 64% were male, and 69% were in practice for ≥10 years. They belong to the following disciplines: medical oncology (41%), radiation oncology (18%), hematology (18%), pediatric oncology (18%), and hematology-oncology (4%). 

Patients’ characteristics are described in Table 1 and Table 2. Of 165 patients, 61% had used telemedicine for the first time. Their median age was 67 years (interquartile range [IQR] 59–75), and the male:female ratio was 1.06:1. Among them, 60% were ≥65 years, 69% were married, and 80% were born in Canada. Overall, 77% were Caucasian, 52% were retired, and 35% had an annual income of  <40,000 Canadian dollars. Of all patients, 68% had a solid tumor, and breast (18%) and lung (16%) were the two most common cancers. Twenty-six percent of patients reported having a curable cancer, 32% had surgery, 27% received radiation, 62% had chemotherapy, and 24% had various other treatments, including endocrine, targeted, and immunotherapy treatment. 

### 3.2. Physicians’ Responses 

Of 25 physicians, 94% were confident in their telemedicine assessment, 58% agreed that telemedicine improved clinical efficiency, and 73% felt that doctor–patient rapport was unimpaired with telemedicine (Table 3). 

### 3.3. Patients’ Responses

Of 165 patients who responded to the survey, 61% had used telemedicine for the first time. Among all patients, 81% agreed or strongly agreed that their needs were met, 83% agreed or strongly agreed with the quality of care, and more than 80% agreed or strongly agreed with their ability to easily communicate with the oncology care team with the available audiovisual setup (Table 4). Of all patients, 88% agreed or strongly agreed about their positive experience with telemedicine consultations. Overall, 95% of patients were willing to participate in another telemedicine consultation. In response to open-ended questions, most patients reaffirmed their support for telemedicine. Selected patients’ comments are provided in Table 5. Among the study participants, 83% of patients vs. 45% of physicians preferred telemedicine to in-person clinic visits (*p* = 0.005). 

### 3.4. Logistic Regression Analysis Results 

On univariate analysis, when various clinical, demographic, and socioeconomic variables were examined, only ages ≥65 compared to ages <65 years were significantly associated with a positive telemedicine experience, with an odds ratio of positive experience of 4.1 (95% confidence interval: 1.2–13.8) (*p* = 0.02). No other variables, including marital status, income, working status, dependent family member, and cancer type, were significantly correlated with a positive telemedicine experience (Table 6).

## 4. Discussion

Our results showed that both patients and physicians had a positive telemedicine experience. The positive patient experience was noted regardless of gender, ethnic background, income, education, work status, type of cancer, treatment goals, and types of treatment. However, age was associated with a differential telemedicine experience. Patients who were of older age had four-fold higher odds of having a positive telemedicine experience compared to younger patients. Because no other factors significantly predicted a positive telemedicine experience, multivariate analysis was not performed. 

Our findings underscore the importance of increasing support for older adults in their communities during their cancer care. Cancer is common in elderly patients. Older age has been associated with an increased risk of comorbidity, frailty, and disability. Furthermore, the likelihood of elderly people living in a single household is high. A traditional health care service requiring long commuting adds an additional burden to elderly patients, especially those with limited support when facing cancer- or treatment-related side effects. The use of the telemedicine service allows elderly patients to stay in their community where they feel safe and have a better quality of life [19,20]. Since the majority of patients were satisfied with their telemedicine consultation, correlation with the other sociodemographic and clinical variables was limited. 

Although physicians prefer face-to-face meetings more than patients do, the majority of the physicians felt that doctor–patient rapport was unimpaired with telemedicine. Cancer care requires a multidisciplinary approach. Telemedicine could facilitate time-efficient multidisciplinary care, as it has the ability to connect multiple health care professionals simultaneously [21]. Furthermore, telemedicine has the potential to facilitate cancer survivorship care, pain and symptom management in the palliative care setting, and participation in clinical trials for rural patients [22,23,24]. Due to the large rural population in Saskatchewan, telemedicine was incorporated into patients’ care by various disciplines several years prior to the COVID-19 pandemic. It has been used by multiple specialists involving more than 440 sites in 147 communities across the province with more than 17,000 appointments, saving patients over 6,000,000 km in travel [25]. Hence, telemedicine has the potential to reduce health-related costs and decrease the health care gap between rural and non-rural populations by reducing travel and waiting time, time away from work, stress of urban commuting, and costs related to travel, parking, and lodging, as well as missed in-person appointments related to travel burden. 

Patient and physician satisfaction is an important factor in ensuring telemedicine is a viable and continuing method of delivering health care. In general, patient satisfaction is closely related to convenience and time and cost saving. Physician satisfaction is more related to clinical efficiency and ease of use [26,27,28]. A systematic review of qualitative research on cancer survivors’ experiences of telemedicine supports the conclusion that telemedicine reduces treatment burden and disruption in cancer survivors’ lives and provides cancer survivors with independence and reassurance [29].

Although telemedicine has enhanced delivery of care in clinical medicine and health promotion, it creates several challenges, including technological issues, limited ability to perform physical examination, and overall effectiveness of the consultation. A scoping review of the perspective of patients and providers on telemedicine in Canada and Australia that contained a large rural population reported that patients and providers perceived reduction in travel time, saving of time and cost related to travel, and improved access to care as potential advantages of telemedicine, whereas lack of face-to-face contact, lack of affordable and reliable technology, poor or inadequate internet access, and poor coordination between urban and rural providers were highlighted by rural patients and their providers as key disadvantages [30]. 

It is important to acknowledge that telemedicine includes both audio and video interaction and encompasses phone assessment, real-time video interaction using a personal device, or video conference at a designated health care center [4,5]. Although telemedicine using a personal device at home would seem to be more convenient than a video link interaction requiring a visit to a local center, it is not suitable for patients who are not familiar or comfortable with technology or who have poor internet service. Furthermore, telemedicine visits using a video link from a rural health care facility to a tertiary care center provide an opportunity for in-person assessment by the health care staff at the local center.

It is worth noting that this study was conducted prior to the COVID-19 pandemic, when telemedicine and virtual care were mostly used for rural residents. The COVID-19 pandemic has transformed the model of care, increasing the prevalence of virtual clinics using audiovisual teleconferencing in multiple settings [10,11,12,31,32,33,34]. The pandemic resulted in a rapid expansion and broadening of telemedicine services, including elimination of geographic restrictions of their use; broadening of eligibility criteria, disciplines, and services while maintaining reimbursement; and improvement in the efficiency and flexibility of consult visits [31,32,33,34,35]. Emerick and others have reviewed the potential advantages and disadvantages of a broader application of telemedicine in pain management, producing findings that are also applicable in cancer care and other disciplines [33]. Early evidence suggests that the rapid implementation of telemedicine in cancer care during the COVID-19 pandemic did not affect quality-of-care indexes, and high rates of patient and care-provider satisfaction were observed [31].

As the COVID-19 crisis has abated, it is important to maintain telemedicine care for a broader population of cancer patients with appropriate guidelines and protocols to ensure the correct balance between face-to-face visits and telemedicine consultations [34]. We believe that a hybrid model that includes an initial face-to-face visit for in-person assessment and establishment of rapport, followed by telemedicine follow-up visits (the frequency and nature of such visits should be tailored to patient needs and convenience) mixed with periodic in-person assessment, will be important in maintaining quality and safe cancer care and patient satisfaction. 

It is important to highlight the limitations of the study, including the relatively small sample size. The survey was developed locally and was not validated. We did not record the number of patients who declined to participate in the study, and hence the response rate cannot be estimated. Of note, telemedicine services were provided at health care facilities closer to patients’ homes rather than at home. Although it is conceivable that a telemedicine consultation at home is potentially more convenient, it is not known whether study results would have been different with the use of virtual visits at home. Lastly, the survey was conducted prior to the COVID-19 pandemic and may not generalize to cancer care during the pandemic.

In summary, our results in the pre-COVID period showed a high rate of positive telemedicine experience in cancer patients and physicians. A high preference for telemedicine was noted among patients compared with physicians. Older patients reported more positive experiences compared with younger patients. No other variable was strongly correlated with positive telemedicine experience. Future longitudinal studies are warranted to explore patients’ and health care professionals’ satisfaction and patients’ reported outcomes with the use of telemedicine. 

## Figures and Tables

**Table 1:** Sociodemographic characteristics of patients.

Variable	N = 165 (%)
Median age (IQR)	67 years (59–75)
Men	84 (51)
Marital status ^a^	
Married or common law relationship	114 (69)
Single	19 (12)
Divorce or separated	8 (5)
Widow	23 (14)
Work Status	
Full time	25 (15)
Part-time	4 (2)
Self-employed	18 (11)
Unemployed	4 (2)
Retired	86 (52)
Disability/sick leave	16 (10)
Home maker	7 (4)
Others	5 (3)
Born in Canada	132 (80)
1–4 children	113 (68)
Ethnic background ^b^	
Caucasian	95/124 (77)
Indigenous	8/124 (7)
Mixed	5/124 (4)
Others	15/124 (13)
Education ^c^	
Elementary school	22/153 (14)
High school	67/153 (44)
Technical/vocational/pre-university degree	34/153 (22)
University degree	28/153 (18)
Income CAD per year ^d^	
Less than 20	13/143 (9)
20–39	37/143 (26)
40–59	28/143 (20)
60–79	15/143 (11)
80 or above	28/143 (20)
Do not know	22/143 (15)

^a^ Information was missing in 1 patient; ^b^ 41 patients did not respond to this question; ^c^ 12 patients did not respond to this question; ^d^ 22 patients did not respond to this question.

**Table 2:** Types of malignant disorders and interventions.

Malignant Disorders and Interventions	N = 165 (%)
Cancer type	
Solid-organ cancer	111 (68)
Blood cancer	27 (16)
Do not know	27 (16)
Five common cancers	
Breast	29 (18)
Lungs	27 (16)
Colorectal	14 (9)
Lymphoma	14 (9)
Prostate	13 (8)
Curable disease ^a^	
Yes	42/163 (26)
No	76/163 (47)
Do not know	43/163 (26)
Surgery	52 (32)
Radiation	44 (27)
Chemotherapy	102 (62)
Other treatments ^b^	40 (24)
Other interventions related to cancer	
Vitamin supplements	31 (19)
Exercise	29 (18)
Relaxation therapy	16 (10)
Special diet	13 (8)
Homeopathy	6 (4)
Yoga	5 (3)
Acupuncture	4 (2)
Using Tele-medicine first time ^c^	101/138 (73)

^a^ Two patients did not respond; ^b^ hormone therapy, immunotherapy, and targeted therapy; ^c^ 27 patients did not respond to this question.

**Table 3:** Physicians’ responses to the survey described in proportion.

Questions	1	2	3	4	5	6
The quality of the image (focus, visual resolution, magnification) was acceptable.	0	0	0	40	40	20
The quality of the audio was acceptable.	0	0	12	18	47	24
I could accurately assess audible symptoms.	10	15	10	40	20	5
The telemedicine clinical exam provided sufficient information.	25	20	25	15	15	0
I am confident in my assessment.	0	0	6	61	33	0
Technical difficulties made this process too time-consuming.	0	20	40	15	20	5
Using telemedicine takes longer than a face-to-face consult.	30	35	10	25	0	0
Overall, the system was accessible and easy to use.	5	0	15	25	35	20
Telemedicine improves clinical efficiency.	5	11	26	42	16	0
My communication with the patient was unimpaired by telemedicine.	0	15	10	30	40	5
I was unable to observe details of the patient’s facial expression and body movements that would have been important in connecting with him/her.	0	40	20	35	5	0
The doctor–patient rapport was unimpaired by the use of telemedicine.	0	16	11	47	26	0
I would have preferred to see this patient in person.	0	30	25	20	10	15

1 = strongly disagree, 2 = disagree, 3 = somewhat disagree, 4 = somewhat agree, 5 = agree, and 6 = strongly agree.

**Table 4:** Patients’ responses to the survey described in proportion.

Questions	1	2	3	4	5	6
Does the ability to provide telemedicine consultation improve your confidence in your cancer care center?	8	1	2	15	29	45
Met your care needs	6	3	1	9	26	55
Overall quality of care provided *	6	1	2	8	29	54
Ability to talk freely over telemedicine	5	2	2	4	20	67
Ability to understand the recommendation made	6	2	2	7	24	59
Quality of the visual image *	6	1	2	6	23	62
Quality of the audio sound *	5	2	1	6	30	56
Overall telemedicine consult experience *	5	3	1	3	29	59

1 = strongly disagree, 2 = disagree, 3 = somewhat disagree, 4 = somewhat agree, 5 = agree, and 6 = strongly agree. * denotes from a negative to positive experience at the scale of 1 to 6.

**Table 5:** Selected comments by the patients who used telemedicine.

Selected Comments
I appreciate being in my home community and not having to drive to Saskatoon for short meetings. I can talk to the doctor and be done quickly.
Extremely happy this is available so we don’t have to make that 2 h trip into the city just for questions, especially if having to make more than one trip a week—very hard when nauseous and no energy
This is a great way to speak with a doctor without having to spend money on gas, time away from work and family. Thank you
We love it
While I appreciate not having to travel to Regina for each telehealth, I would not mind meeting face to face with my oncologist a few times per year. I have met with him face to face only once in over 2 years; the initial discussion of my diagnosis and treatment plan. Alternatively, maybe a specific visit with my family doctor (hands on + look me in the eyes) and then he and my oncologist and I could have a 3-way call.
Telehealth is wonderful, saves a lot of trips to the city
It worked great
We appreciate that we do not have to travel a great distance. We really appreciate being able to have these telemedicine consultations.
Excellent and we don’t have to drive for 3 h to get to the Doctor in Saskatoon.
Courteous service
Everything seems very efficient from the experience I have.
Excellent!
When working full time it makes it difficult to go to the city every time. Telehealth appointments are convenient for my lifestyle. Sometimes I feel the telehealth might not be as thorough since the visual of the patient might not be easy. If the doctor would need to examine the patient.
They work well for me and I appreciate not having to travel to Saskatoon since I no longer drive and it costs me 100$ each way to hire a driver
An excellent alternative for people living in rural SK. Having the ability to receive chemotherapy or consult with your doctor in your home community significantly reduces stress during a very difficult time. Thank you for this advancement in health care.
I wish that more specialists would participate in Telehealth. The $$$ would be very beneficial.
I think it’s amazing that I don’t have to travel 4+ hours for an appointment that usually lasts a few minutes. Thank you so much for offering this.
Telehealth works well for checkup consults. Physical checkups are scheduled as required. Please keep up great program
All really good, keep it up
Great services especially for those of us that do not live in Saskatoon and Regina
Thankful telemedicine is available. We don’t have to made the drive to Saskatoon for our appointments.
Teleconference very nice in winter months to save having to drive in cold/stormy weather
Picture quality is poor, so it’s very hard to see facial expressions and body language.
Great the way it is.

**Table 6:** Univariate analysis of association between a positive telehealth experience and various socioeconomic and demographic factors.

Variable	Odd Ratio (95% Confidence Interval)	*p* Value
Age ≥ 65 years	4.1 (1.2–13.8)	0.02
Women	1.07 (0.3–3.12)	0.90
Single or widowed	1.23 (0.25–5.95)	0.79
Non-Caucasian	2.01 (0.54–7.42)	0.29
Working	2.8 (0.91–8.4)	0.07
Dependent Children	3.0 (0.37–23.5)	0.31
Non-university education	3.4 (0.43–27.2)	0.24
Income < 80 K	1.6 (0.46–5.5)	0.45
South Center	1.4 (0.43–4.3)	0.58
Blood cancer	1.75 (0.50–6.10)	0.38
Non-curable cancer	3.11 (0.65–14.90)	0.16
No surgery	1.7 (0.47–6.6)	0.40
Radiation	2.2 (0.72–6.8)	0.16
Chemotherapy	1.6 (0.48–5.3)	0.44

## Data Availability

The data presented in this study are not publicly available. Data access will require approval form the University of Saskatchewan Biomedical Ethics Board and Data Access Committee of the Saskatchewan Cancer Agency.
